# Late Holocene anthropogenic landscape change in northwestern Europe impacted insect biodiversity as much as climate change did after the last Ice Age

**DOI:** 10.1098/rspb.2021.2734

**Published:** 2022-06-29

**Authors:** Francesca Pilotto, Alexis Rojas, Philip I. Buckland

**Affiliations:** ^1^ Environmental Archaeology Laboratory, Department of Historical, Philosophical and Religious studies, Umeå University, Umeå, Sweden; ^2^ Integrated Science Laboratory (Icelab), Umeå University, Umeå, Sweden; ^3^ Norwegian Institute for Nature Research (NINA), Oslo, Norway

**Keywords:** Coleoptera, fossil beetles, palaeoentomology, biotic transitions

## Abstract

Since the last Ice Age (*ca* 115 000–11 700 years ago), the geographical ranges of most plants and animals have shifted, expanded or contracted. Understanding the timing, geographical patterns and drivers of past changes in insect communities is essential for evaluating the biodiversity implications of future climate changes, yet our knowledge of long-term patterns is limited. We applied a network modelling approach to the recent fossil record of northwestern European beetles to investigate how their taxonomic and trait composition changed during the past 16 000 years. We found two major changes in beetle faunas 4000–3500 and 10 000–9500 years ago, coinciding with periods of human population growth in the Late Holocene and climate warming in the Early Holocene. Our results demonstrate that humans have affected insect biodiversity since at least the introduction of agropastoralism, with landscape-scale effects that can be observed at sites away from areas of direct human impact.

## 1. Introduction

Between the Last Glacial Maximum and the present day, climatic fluctuations, increasing human populations and social changes have significantly influenced landscapes and biodiversity globally [1]. Arctic-alpine and glacial plant and phytoplankton species were, for example, driven to regional extinction in the Baltic region during the Younger Dryas–Holocene transition, *ca* 11 700 years before present (BP) [2]. In addition to natural drivers, humans have extensively modified natural biomes, especially since the introduction of agriculture [3,4]. Disentangling the causes of species geographical shifts and extinctions is often difficult, as exemplified by the debate on the relative importance of humans as drivers of megafaunal extinctions in the Late Quaternary [5,6].

Beetles (Coleoptera) are the most species-rich animal order on Earth [7]. They are extremely valuable ecological indicators, as individual species have different climatic, ecological and physiological requirements, and numerous species are restricted to specific habitats [8]. Consequently, major transitions in species composition indicate major changes in the landscape and/or climate. Indeed, previous studies have demonstrated the capacity of fossil beetles for describing past temperature changes [9,10], and changes in fossil beetle assemblages have often been used as a proxy for past environmental changes at the local scale [11]. They have also recently been used to interpret the drivers and consequences of pollen diversity changes over millennial timescales [12], but they have rarely been studied in terms of their own biodiversity. Understanding the broader picture of past species turnover and its drivers is essential for understanding the potential of a future mass extinction event [13].

**Figure 1.**
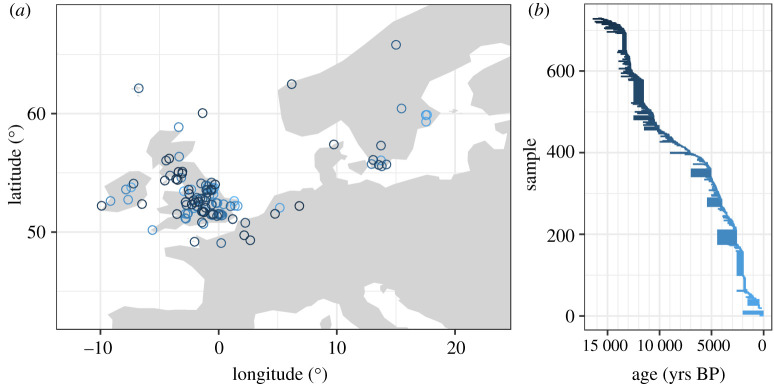
Geographical and temporal scope of the study: location of the 729 samples at 145 sites (*a*). The colour of the dots reflects the age ranges of the samples (*b*). (Online version in colour.)

Here we investigate the Late Quaternary record of fossil beetles [14,15]. The data comprises occurrences of 1225 species in 729 samples at 145 deposits from northwestern Europe, dating from 16 000 BP to the present (figure 1). We analysed both taxonomic composition and ecological traits describing species-specific habitat affinities (electronic supplementary material, table S1), by creating species- and trait-based multilayer networks [16], where layers represent ordered time intervals and nodes represent taxa, or traits, and samples [17]. We then applied a network modelling approach, i.e. multi-level clustering, to: (i) identify the major biotic transitions in beetle faunas, i.e. major changes in their biodiversity, as reflected in taxonomic and trait composition, during the Late Quaternary; and (ii) evaluate whether such transitions are spatially and temporally concurrent between species- and trait-based analyses. This multilayer network approach was recently used to identify biotic transitions in fossils [17], but has not previously been applied to fossil insect data.

## 2. Methods

### (a) Data

We analysed the Quaternary fossil record of beetles from the BugsCEP constituent database (www.bugscep.com, downloaded on 3 December 2020) [15] of the open access Strategic Environmental Archaeology Database (SEAD; www.sead.se) [14]. These data have been gathered from over 1400 published studies in both scientific journals and consultancy reports, including commercial contract archaeology and environmental impact reports, which are not part of the general scientific literature. The database is global, but includes data mostly from northwestern Europe, with other clusters in Iceland, Greenland, North America and the Mediterranean area (https://browser.sead.se/palaeoentomology; [15]). For this study, we restricted our analysis to the large concentration of well-dated sites in northwestern Europe, and especially the British Isles. The geographical and temporal scope of the fossil insect data used in this study (after the filtering described below) is shown in figure 1. References to the source publications are available through BugsCEP and SEAD.

Sites in SEAD are classified as either archaeological or geological, with each site containing data from one or more sediment samples. Archaeological sites are locations that have most likely been directly influenced by past human activities, at or near archaeological excavations. Geological sites, hereinafter ‘natural deposits’, are either peatbogs or lake sediments located at some distance in space or time from known archaeological sites. These sites are assumed, on the basis of their source publications, to have no *direct* human impact (until proven otherwise), and reflect background landscape changes recorded in sediments deposited through natural processes. Over time, and especially in recent centuries, the degree to which human impact on the landscape is reflected in these deposits is expected to increase. For this study, we used only data from natural deposits, which provide proxy evidence for climate and environmental change. Sample dates are expressed as a range of years BP and derived through a wide variety of dating methods ranging in precision and accuracy from modern radiocarbon dating to stratigraphic association of undated samples with dated layers [18]. A large number of dates are also interpolated between radiometric dates using age-depth models. Buckland [19] provides an overview of the dating evidence and problems inherent in its nature, and the details of individual samples are available in the database [14,15]. In our analyses, we used 500-year bins (see §2b) and we applied two filtering criteria to the data to reduce the risk of inheriting problems from poor dating. First, in order to maintain a complete temporal sequence, we excluded samples with a temporal mid-point (middle point between maximum and minimum value) larger than 16 000 years. The excluded samples represent the oldest records in the data and create disconnected nodes and sparse layers in our ordered temporal framework. Second, we excluded samples with an age range larger than 2000 years, as samples with large age uncertainty would obscure temporal structures in the multilayer network framework (see §2c). The majority of samples fulfilling these criteria are located in northwestern Europe and especially the British Isles. The geographical focus of this study was thus limited to ensure that samples would collectively reflect a continuous record of changes from the end of the last Ice Age to the present day (figure 1).

Taxonomic resolution in the database varies: 69% of the occurrences in the database are species, 27% are genera (or species aggregates) and 4% are families. Here, we used only taxa identified to species level, thus ensuring the highest level of resolution in both taxonomy and ecological implications. Each species in SEAD is associated with ecological traits (EcoCodes) [20] that describe its affinity for one or more of 22 habitat types. These traits are derived from the BugsCEP database [15] and in part based on Koch [21,22] but adapted for use in palaeoecology [20]. The BugsCEP trait classification system uses 22 categories designed to help in the analysis of insects as proxies for landscape change and human activities. The habitat traits vary in scale from specific indicators (of e.g. deciduous wood or woodland, dung) to broader landscape components such as ‘wood and trees’ or ‘open wet habitats’ [20]. As such, they provide scope for tracking (the insect's eye view of) broad-scale environmental changes and identifying some of the more nuanced results of human activity (e.g. grain storage). In this study, no ectoparasites were present in the samples, and thus only 21 of the traits are used. See the electronic supplementary material, table S1 for a full list of traits and their descriptions. We created two datasets: a sample × species matrix, containing species presence data, and a sample × trait matrix, containing the relative number of species (expressed as decimal number between 0 and 1) associated with the different traits in the samples. The filtered dataset comprises a total of 145 sites, 729 samples, 1225 beetle species and 21 426 individuals.

### (b) Network representations

We created two different multilayer network representations from the filtered datasets, one based on the taxonomic dataset and one based on the ecological trait dataset (electronic supplementary material, figures S1–S3). This network framework was recently developed to describe macroevolutionary patterns in the Phanerozoic fossil record of benthic marine animals [17]. It explicitly represents temporal constraints in the underlying data as ordered layers assembled into a single network and uses state nodes to distinguish the same taxa occurring in multiple layers. Because the physical node representing a given taxon comprises as many states nodes as time intervals where this taxon occurs, our multilayer network representation captures higher order dynamics, i.e. beyond pairwise relationships, and allows us to delineate overlapping assemblages [17]. In contrast with standard unipartite and bipartite networks, our multilayer network framework allows us to capture the complex spatio-temporal organization in the fossil record and thus reflects changes in insect biodiversity over time.

#### (i) Taxonomic network

We created a multilayer network representation of the taxonomic dataset (filtered sample × species matrix). Layers in this network representation correspond to ordered 500-year time intervals. This temporal resolution is sufficient to capture broad-scale changes since the Last Glacial Maximum without high-resolution details confounding the analysis and has been previously used in Quaternary studies [23]. We established that the results are independent of the selected temporal resolution by repeating the analyses using layers of different time length. The modular structure of the assembled network also emerges in network representations created using time bins ranging between 400 and 500 years. By selecting a bin length in the top of this range, we used a larger number of samples from the underlying data (electronic supplementary material, figures S4 and S5). Our representation is a temporal network comprising two sets of physical nodes, beetle taxa and temporally explicit samples. Because physical nodes representing beetle taxa can occur in multiple layers, we divided these physical nodes into state nodes [16], with one state node per layer per given taxon. Samples are temporal-dependent physical nodes and thus they are represented by a single-state node [17]. Each layer in this input representation can be considered a bipartite network [24] with nodes representing beetle taxa and layer-dependent samples, linked together by unweighted edges describing their connectivity. The taxonomic network comprises 1954 physical nodes (*n*), including 729 samples and 1225 taxa, joined by 9798 intralayer links (*m*), distributed into 32 layers (*t*).

#### (ii) Trait network

We created a multilayer representation of the ecological trait dataset (filtered sample × trait matrix). Similar to the taxonomic network above, layers in the trait network represent ordered 500-year time intervals. A total of 21 physical nodes were created to represent the individual traits (electronic supplementary material, table S1). Similar to the taxonomic network, physical nodes representing ecological traits can occur in multiple layers and are divided into state nodes, with one state node [16] per layer in which a given trait occurs. Samples are layer dependent and are represented by a single-state node. Because beetle taxa can be associated with one or more traits, we create weighted edges describing the connectivity strength between samples [24] and traits. Consequently, each layer in the trait network consists of traits and layer-dependent samples linked together by weighted edges. The trait network comprises 750 physical nodes (*n*), including 729 samples and 21 traits, joined by 4820 links (*m*), distributed into 32 layers (*t*).

### (c) Network clustering

We used the map equation framework [16] to cluster the two input networks. The map equation models information spreading on a given network with a random walk and capitalizes on the duality between two processes, encoding information using fewer bits and finding regularities, to reveal the network's modular structure. A random walk is the simplest model for studying information flow in a network [25], and in our analysis, it describes a succession of random steps between samples and taxa that are connected by a link. In the multilayer framework, interlayer relationships are modelled based on the intralayer links in the network. Intralayer links are unweighted connections between nodes representing samples and taxa in the taxonomic network, and weighted connections between samples and traits in the trait network. In practice, interlayer links are created by relaxing the constraint of the random walker moving in a given layer to neighbouring layers where the same taxon or trait occurs (see relax rate below). Besides the objective function that evaluates the quality of a network partition [25], the map equation framework includes a search algorithm that optimizes this function over different solutions to provide an optimized multi-level solution for the input networks [16].

We obtained reference solutions for the taxonomic and trait networks using the following Infomap [16] arguments: N = 500, i = multilayer, multilayer-relax-rate = 0.25, multilayer-relax-limit = 2. The multilayer-relax rate (*r*) is the probability to relax the constraint of a random walker moving only in the current layer to a random layer where the same taxon or trait occurs. Following a previous multilayer-network analysis of fossil records, we used a relax rate *r* = 0.25 that is large enough to enable interlayer (temporal) interdependencies but small enough to preserve intralayer information [24]. However, we show that the modular structure of the assembled networks is robust to a range of relax rates (electronic supplementary material, figure S6).

In our multilayer model, a random walker within a given layer moves between samples and taxa, or samples and traits, guided by intralayer links with probability (1 − *r*), and it relaxes to a random layer with a probability *r*. The relax limit *(l*) represents the number of adjacent layers in each direction to which a random walker can relax, guaranteeing the temporal ordering of layers. In our model, the relax limit is a global parameter based on the accepted age range when filtering the data: with layers representing 500-year time intervals, an accepted age range of 2000 years translates into a relax limit *l* = 2. In this way, our clustering procedure guarantees that a random walker will relax only to neighbouring layers without exceeding the age limits of the samples in the filtered dataset. In general, the selection of the number of adjacent layers used to relax the random walker on the ordered layers is a compromise between the loss of temporal resolution when filtered samples have a large age range, which translates into *l* greater than 1, and the loss of samples when shortening the accepted age range in the filtered data. In summary, a relax limit *l* = 1 is the minimum value that enables interlayer connections but it reduces the number of samples in the filtered data. Here, we use a relax limit *l* = 2 because it is still short enough to enable the temporal ordering of the layers and allows us to use more samples from the underlying fossil data.

Infomap modules in the reference solutions are clusters of both taxa and samples, and traits and samples. They are labelled from the largest to the smallest based on their size (number of nodes). Here, we named trait-based modules from TM1 to TM8 and species-based modules from SM1 to SM3 to indicate, from older to younger, their temporal distribution. The network visualization in the electronic supplementary material, figures S1 and S2 shows the nested hierarchical structures of the reference solutions. Inter-module links in hierarchical levels 1 and 2 reveal the temporal structuring of modules.

### (d) Robustness assessment

We measured the robustness of different aspects of the optimized Infomap solution (i.e. reference solution) by using bootstrap replicates. We created 100 replicate networks with weighted links derived from a normal distribution with mean 1 and a variance that prevents negative values. We clustered the replicate networks using the same Infomap arguments used to obtain the reference solution (N = 500, i = multilayer, multilayer-relax-rate = 0.25, multilayer-relax-limit = 2), and compared the bootstrap partitions against the reference solution.

First, using the significant clustering [26], we identify the subset of nodes that are significantly assigned to modules in the reference solution. We called this subset of nodes the significant core of the module. In addition, we identified pairs of modules that are mutually significant. Specifically, two successive modules in the reference solution are mutually significant if their cores are clustered together in less than 5% of all bootstrap partitions. In this case, we define the module transition as abrupt. By contrast, if their cores are clustered together in greater than or equal to 5% of all bootstrap partitions, two successive modules are mutually non-significant, and the module transition is gradual, with substantial overlap. Overall results indicate that the taxonomic transition observed at 4000–3500 BP is abrupt but the one at 10 000–9500 BP is gradual (electronic supplementary material, figure S1). Four module interactions in the trait network represent temporal transitions; one gradual transition at 12 000 BP, and abrupt transitions at 10 000, 8000, and 6500 BP (electronic supplementary material, figure S2). Second, we estimated the significance of the reference solution at the layer level. Specifically, we compared each layer in the reference solution against the corresponding layer in each bootstrap partition and computed the proportion of paired comparisons with a Jaccard similarity higher than 0.70. This procedure for estimating module robustness is described in Calatayud *et al*. [27]. Overall, modules are less robust near the observed transitions (electronic supplementary material, figure S3).

### (e) Climate reconstructions

**Figure 2.**
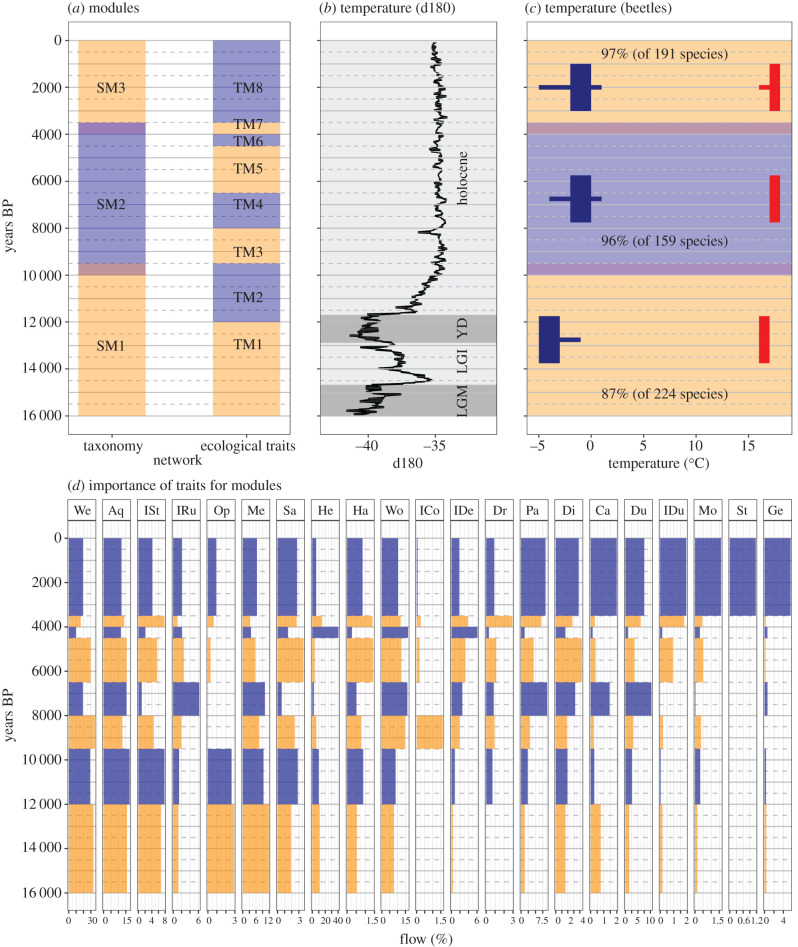
(*a*) Results of the network analysis based on the taxonomy (modules SM1–3) and ecological traits (modules TM1–8). (*b*) Relative temperature changes based on oxygen isotope data from Greenland ice cores [28] are shown as a reference. LGM, Last Glacial Maximum; LGI, Late Glacial Interstadial; YD, Younger Dryas. (*c*) Beetle-based MCR reconstruction [9] of the mean temperature of the coldest (TMin, blue) and warmest (TMax, red) months, based on the temperature tolerances of climate reference species occurring in modules SM1–3. The percentage of climate-sensitive species in the final MCR result is shown for each module (see methods). Bars show the jackknife ranges and indicate the robustness of the reconstruction. (*d*) Changes in trait importance for the modules, defined as the per cent flow volume resulting from the network analysis [25]; colours delineate trait-based modules; note the different scales on the *x*-axes. See the electronic supplementary material, table S1 for a description of the traits: We, wetlands/marshes; Aq, aquatics; ISt, indicators–standing water; IRu, indicators–running water; Op, open wet habitats; Me, meadowland; Sa, sandy/dry disturbed/arable; He, heathland and moorland; Ha, halotolerant; Wo, wood and trees; ICo, indicators–coniferous; IDe, indicators–deciduous; Dr, dry dead wood; Pa, pasture/dung; Di, disturbed/arable; Ca, carrion; Du, dung/foul habitats; IDu, indicators–dung; Mo, mould beetles; St, stored grain pest; Ge, general synanthropic. (Online version in colour.)

We performed a climatic reconstruction for each module that we obtained from the taxonomic network analysis, based on the species composition in each module. We performed climatic reconstructions using the mutual climatic range (MCR) method [9], as implemented for 437 beetle species in the BugsCEP software [20]. Specifically, we used the thermal envelopes (i.e. thermal tolerances) of the species clustered in a module to obtain the area of greatest climatic overlap, which represents the most probable thermal range for the module. The percentage of species that forms the area of greatest climatic overlap (in climate space) for a module provides an indication of the degree of climate change, or inter-site variability, within that module (the lower the percentage overlap, the greater the climate change) [20]. The thermal envelope of a species is defined by its modern thermal distribution (see Buckland [20]). Herbivorous species are excluded from the MCR computation as their geographical distribution may be dependent on the presence of host species rather than climatic factors [10]. The MCR method was run on two climatic variables: the mean temperature of the warmest month (TMax) and the mean temperature of the coldest month (TMin). Results are shown as TMax and TMin ranges. We ran a jackknifed MCR routine in which each and every taxon was removed individually from the MCR calculations, to evaluate the robustness of the MCR reconstructions [20]. As reference, in figure 2, we show the relative temperature changes based on an independent dataset: the oxygen isotope data from Greenland ice cores [28].

### (f) Importance of traits for the trait-based modules

The importance of the traits for the modules is defined as the amount of flow volume, i.e. the probability that the random walker visits a trait, resulting from the network analysis [25]. For each module, we standardized the flow volume of each trait by the total volume for that module by computing the per cent flow per module per layer. To evaluate the changes in trait importance through time, we displayed these values of per cent flow per trait per module in figure 2*d*. To identify the significant associations between traits and modules, we ran an indicator value (IndVal) [29] analysis, based on the per cent flow per trait per layer (i.e. 500 year bin), following the same approach as explained above for the per cent flow per trait per module. We ran the IndVal analysis for the groups of trait-based modules that reflect the species-based modules, using the R package ‘*indicspecies*’ [29].

## 3. Results and discussion

The optimized clustering of the species- and trait-based networks delineate temporal modules, i.e. assemblages that sequentially dominate the landscape and shift dominance patterns over time (figure 2*a*). The species-based network is best described by three modules with a gradual transition (taxonomic overlap after bootstrap: greater than 5%) occurring at 10 000–9500 BP and an abrupt transition (overlap after bootstrap: less than 5%) at 4000–3500 BP. The trait-based network confirms these two major events in the fossil record of beetles by showing an abrupt transition at 9500 BP and complex module interactions between 4500 and 3500 BP (electronic supplementary material, figure S2). Moreover, the ecological trait network indicates additional transitions at 12 000, 8000 and 6500 BP. The differences between modules can be explained in terms of both the climatic and environmental dependencies of their species (figure 2).

### (a) Late Glacial period

The species-based module SM1 (16 000–9500 BP) extends from the cold Last Glacial Maximum to beyond the Younger Dryas. This period is primarily characterized by cold winter and summer temperatures, as indicated by the MCR [9,20] temperature reconstruction for the species clustered into this module (figure 2*c*). The proportion of species falling into the area of maximum overlapping climate space is lower in SM1 (87%), than SM2 (96%) and SM3 (97%). This most likely reflects the large degree of temperature variation captured by this model. Indeed, our analysis could not resolve the Last Glacial Maximum, the warmer Late Glacial Interstadial and the colder Younger Dryas (figure 2*b*) as separate modules.

The trait-based modules TM1–2 show a dominance of beetle species that are associated with open wet habitats, wetlands and meadowland (table 1). The early phase of this period (TM1) is characterized by species associated with open wet habitats (table 1), as well as meadowlands (figure 2*d*). This trait configuration reflects the presence of mobile environments, especially at the glacial margins, with water over the permafrost, during the Late Glacial and Last Glacial Maximum [30]. The termination of this phase coincides with the end of the Younger Dryas as closely as our 500 year resolution allows.

**Table 1.** Indicator value (IndVal) analysis, based on the importance of traits for modules (i.e. relative flow volume resulting from the network analysis) of the samples belonging to each module. (Significant (*p* < 0.05) associations between traits and modules are shown. The analysis was performed for the groups of trait-based modules that reflect the three species-based modules (figure 2*a*).)

	trait	importance (IndVal)	*p*
TM1-TM2	open wet habitats	0.77	0.017
(16 000–9500 BP)	meadowland	0.70	0.004
	wetlands/marshes	0.66	0.004
TM3-TM7	wood and trees	0.66	0.014
(9500–3500 BP)			
TM8	general synanthropic	0.94	0.001
(3500–0 BP)	indicators–dung	0.89	0.001
	carrion	0.84	0.002
	mould beetles	0.83	0.013
	pasture/dung	0.77	0.002
	dung/foul habitats	0.74	0.005
	disturbed/arable	0.69	0.047
	stored grain pest	0.66	0.011

The second phase of this period (TM2) spans between 12 000 and 9500 BP. This phase sees an increase in the proportion of beetle species associated with sandy/dry disturbed/arable lands and halotolerant species. These open, sun-exposed landscapes, with nutrient-rich sediments, and the beetles species they support, are now more common on ploughed agricultural lands and coastal environments [31].

### (b) Early to mid-Holocene

We identified a major biotic transition at 10 000–9500 BP in both species (SM1 to SM2) and trait-based (TM2 to TM3) networks (figure 2*a*). The event is concurrent with the transition to stable warmer conditions during the Early Holocene (figure 2*b*), as shown in our MCR temperature reconstruction (figure 2*c*). The statistical significance of the modules drops at their boundaries, reflecting instability in the assembled networks across biotic transitions (electronic supplementary material, figure S3). For instance, the drop in significance at 10 000–9500 BP is likely to reflect the expansion of trees from glacial refugia in the Early Holocene [32,33]. Indeed, the trait-based module TM3 and TM4 (9500–8000 and 8000–6500 BP) are dominated by beetle species linked to wood and trees, and especially conifers in TM3 (table 1; figure 2*d*). This is in line with pollen-based forest reconstructions, which show that forest cover expanded in Europe from 11 000 to *ca* 8000 BP, reaching a maximum at 8000–6000 BP, and then gradually decreased [34].

The trait-based module TM4 is also characterized by species linked to pasture/dung, dung/foul habitats, and supports earlier interpretations of the insect evidence for a pasture-woodland-like landscape in the British Isles [35].

### (c) Mid and Late Holocene

The trait-based analysis delineates a biotic transition in the fossil record of beetles at 6500 BP (TM4 to TM5; figure 2*a*). This abrupt change coincides with the Mesolithic–Neolithic transition in northern Europe, characterized by increased human populations, changes in land use and the introduction of pastoralism and agriculture [36]. This biotic transition also coincides with a shift in the assembly of plants and mammals from aggregate to segregated species co-occurrence structures, attributed to increasing anthropogenic disturbances [37]. Insect data from archaeological sites (not included in this analysis, and including earlier grain pest records) suggest a transition from extensive, low impact land use, through increasingly intensive local impacts, to extensive high impact land use [38]. The timing of these land use transitions varies across Europe, being generally earlier in the southeast and later in the north and west, but probably occurring in multiple waves of colonization and technological exchange as agriculture and pastoralism become the dominant modes of subsistence [38]. The date of the transition from TM4 to TM5 suggests that the network analysis is, through the fossil beetle record, identifying the beginning of extensive landscape changes in northwestern Europe towards agropastoral landscapes.

The ecological trait network also shows two transitions between TM5, TM6 and TM7 (4500 and 4000 BP; figure 2*a*). However, the peak of heathland and deciduous trees in TM6 may be an artefact of several samples with poorly preserved insect remains from the peat underneath a Neolithic trackway [39] and targeted sampling of preserved trees which do not provide a landscape reconstruction. After peaking *ca* 4000 BP, with an associated peak of dry dead wood, deciduous trees decline and species associated with pasture, dung/foul habitats and carrion begin to dominate, suggesting the increased presence of animals in the landscape (figure 2*d*). The trait signals of TM5 to TM8 confirm previous pollen-based land cover reconstructions for the British Isles that showed accelerated deforestation after 4000 BP [34], coinciding with increasing human populations [36]. An acceleration of vegetation change during the Late Holocene (4200 BP—onwards) has been recorded both globally and for Europe [40], with the conversion of forest into open agricultural land [23].

The taxonomic and trait-based analyses delineate a biotic transition at 4000–3500 BP. This period is characterized by the arrival of or significant increases in the abundance of species associated with synanthropic habitats, stored grain, mould, carrion, pasture/dung and dung/foul habitats (table 1; figure 2*d*). Charcoal analyses show that a transition at 3500 BP also indicates a progressive increase in fire frequency in Europe that continues into the Late Holocene [41].

## 4. Conclusion

Our analysis of beetle biodiversity, as indicated through taxonomic and trait composition, in northwestern Europe over the past 16 000 years reveals two major biotic transitions, at 10 000–9500 and 4000–3500 BP. The first transition coincides with a period of known climatic and environmental changes, and the second with a period of increasing human influence on the landscape. Our results are in line with those previously obtained with other proxies [33,34,37] and show that fossil beetles can reliably track climatic and environmental changes beyond the local scale (e.g. [11]), at large spatial and temporal scales. The beetle data also provide additional information on the presence of grazing animals in the landscape that are not easily visible through other proxy data sources (e.g. [8,12,18,35]). The appearance of traits related to agropastoralism also demonstrates that humans have impacted the biodiversity of beetles in northwestern Europe for at least 6500 years.

Regardless of the driving forces, our study suggests that large-scale insect biodiversity, taxonomic and trait patterns responded differently to major environmental and climatic changes during the Holocene. A transition in the taxonomic network at 10 000–9500 BP identifies the stabilization of temperatures in the Early Holocene, whereas the majority of transitions in the trait-based network respond to changes in the landscape. The decoupling of taxonomic from functional turnover has also been found in mammal assemblages over the past 21 Myr [42]. Our trait analysis identifies five further biotic transitions in addition to those identified in the taxonomic analysis. These seem to reflect more subtle changes in the landscape. We therefore agree with Blanco *et al*. [42] on the value of combining taxonomic and trait-based analyses in palaeobiological studies to achieve a more robust understanding of past environmental changes, and which can be of more use to conservation policy than taxonomic studies alone [43]. For example, when evaluating the success of rewilding or restoration projects [44], it is important to look at not only the resulting changes in the diversity of insect and plant species, but also the habitats indicated by the insects themselves. The results of this study, and others cited herein, also strongly suggest that the use of relatively recent pre-industrial baselines as targets for restoring ‘natural’ landscapes and biodiversity [45] is highly questionable.

We found that the major biotic transitions occur simultaneously across the studied sites (within the limits of our 500 year resolution), indicating no geographical structures in both biodiversity and trait change, at the largest scales of our multi-level analysis (electronic supplementary material, figure S3). Our results thus show that, in the studied area, large-scale processes, especially past climate and environmental change and human impacts, override biogeography in driving beetle biotic transitions. This has been previously observed in the fossil record of marine invertebrates [17].

We have demonstrated the unique insights provided by fossil beetles into past human activities, such as the introduction of animals and agriculture, and the modification of environments beyond the bounds of natural processes. Furthermore, in the light of predictions for future climate and landscape changes [46,47], our findings suggest that beetle assemblages are likely to experience further biotic transitions in the near future. Under landscape or climate changes, the survival of any species is dependent on the capacity of its populations to migrate to suitable areas [48]. Our results suggest that human modification of the landscape, as identified in previous studies [1,3,12,33,36,37], can have dramatic effects on insect biodiversity over long timescales. If the future rate of landscape change exceeds the capacity of insects to adapt, or if there are no longer any suitable areas to migrate to, then species will undoubtedly become extinct [49].

## Data Availability

The fossil beetle data and trait associations are available from the Strategic Environmental Archaeology Database (www.sead.se) [14]. Note that our analysis uses a subset of these data as explained in the methods section. This subset of data, the assembled taxonomic and trait networks, the reference solutions, and the source code for reproducing the figures and IndVal analysis are available at a GitHub repository: https://github.com/humlab-sead/Pilotto_Rojas_Buckland_2022. The Infomap software package was used for clustering the assembled networks into multi-level partitions. It is freely available as a client-side web application at https://www.mapequation.org. Data is also provided in the electronic supplementary material, figures S1–S6 and table S1 [50].
